# Evaluation of a therapy for Idiopathic Chronic Enterocolitis in rhesus macaques (*Macaca mulatta*) and linked microbial community correlates

**DOI:** 10.7717/peerj.4612

**Published:** 2018-04-11

**Authors:** Joshua M. Taylor, Erik L. Clarke, Kate Baker, Abigail Lauder, Dorothy Kim, Aubrey Bailey, Gary D. Wu, Ronald G. Collman, Lara Doyle-Meyers, Kasi Russell-Lodrigue, James Blanchard, Frederic D. Bushman, Rudolf Bohm

**Affiliations:** 1 Division of Veterinary Medicine, Tulane National Primate Research Center, Covington, LA, United States of America; 2 Department of Microbiology, University of Pennsylvania School of Medicine, Philadelphia, PA, United States of America; 3 Division of Gastroenterology, Hepatology, and Nutrition, The Children’s Hospital of Philadelphia, University of Pennsylvania School of Medicine, Philadelphia, PA, United States of America; 4 Department of Microbiology and Immunology, Lineberger Comprehensive Cancer Center, University of North Carolina at Chapel Hill, Chapel Hill, NC, United States of America; 5 Division of Pulmonary, Allergy and Critical Care, Department of Medicine, University of Pennsylvania School of Medicine, Philadelphia, PA, United States of America

**Keywords:** Chronic colitis, 16S metagenomic sequencing

## Abstract

Idiopathic chronic enterocolitis (ICE) is one of the most commonly encountered and difficult to manage diseases of captive rhesus macaques (*Macaca mulatta*). The etiology is not well understood, but perturbations in gut microbial communities have been implicated. Here we evaluated the effects of a 14-day course of vancomycin, neomycin, and fluconazole on animals affected with ICE, comparing treated, untreated, and healthy animals. We performed microbiome analysis on duodenal and colonic mucosal samples and feces in order to probe bacterial and/or fungal taxa potentially associated with ICE. All treated animals showed a significant and long-lasting improvement in stool consistency over time when compared to untreated and healthy controls. Microbiome analysis revealed trends associating bacterial community composition with ICE, particularly lineages of the Lactobacillaceae family. Sequencing of DNA from macaque food biscuits revealed that fungal sequences recovered from stool were dominated by yeast-derived food additives; in contrast, bacteria in stool appeared to be authentic gut residents. In conclusion, while validation in larger cohorts is needed, the treatment described here was associated with significantly improved clinical signs; results suggested possible correlates of microbiome structure with disease, though no strong associations were detected between single microbes and ICE.

## Introduction

Gastrointestinal disease is one of the most significant causes of morbidity and mortality in captive nonhuman primates worldwide (Ribbons et al., 1997; Blackwood et al., 2008; Wilk et al., 2008; Broadhurst et al., 2012; Ferrecchia & Hobbs, 2013; Prongay, Park & Murphy, 2013), at the Tulane National Primate Research Center (TNPRC) costing at least $500,000 annually. A three-year retrospective study performed at TNPRC revealed an average annual incidence of diarrhea of 73.95 per 1,000 animals—approximately 300 cases per year (involving both indoor housed and outdoor housed animals). Of the 300 cases, 67 animals required treatment on more than one occasion (range: 2–5 presentations). Individuals were hospitalized for a mean of 42 days (range 1–225 days; median 31 days). Beyond the immediately measurable effect on resources, the potential loss of genetically valuable breeding stock and the removal of animals from their social groups for diagnosis and treatment exerts a negative effect on a colony’s genetic diversity and wellbeing (Snyder-Mackler et al., 2016).

Idiopathic chronic enterocolitis (ICE) of rhesus macaques (*Macaca mulatta*) is frequently reported as a particularly challenging condition to manage (McKenna et al., 2008; Broadhurst et al., 2012; Ardeshir et al., 2013; Ferrecchia & Hobbs, 2013). Similar to human forms of chronic colitis, such as Crohn’s disease or ulcerative colitis, macaque ICE is characterized by chronic relapsing episodes of soft or liquid stool, the histological presence of inflammatory lesions in the intestinal tract, and a lack of identifiable pathogens (Sestak et al., 2003; Damman et al., 2012; Rice et al., 2013). Often these cases are intractable to conventional therapy and result in euthanasia due to chronic diarrhea, weight loss, decline in body condition, failure to thrive, and development of sequelae such as amyloidosis (Prongay, Park & Murphy, 2013; Rice et al., 2013). While gross lesions may not be visible endoscopically, ICE biopsy specimens are typically characterized as a lymphoplasmacytic enterocolitis on histologic evaluation with a variety of observed changes including crypt hyperplasia and/or abscesses, goblet cell depletion, villous atrophy and/or tufting, and variable expansion of the enteric lamina propria with inflammatory infiltrates (Blackwood et al., 2008; Wilk et al., 2008; Ardeshir et al., 2013).

While many different treatment modalities have been studied, few have demonstrated long term success (Ribbons et al., 1997; Wilk et al., 2008; Broadhurst et al., 2012). Dietary supplements such as coconut, antibiotics (tylosin), oral fecal bacteriotherapy, inulin, iNOS inhibitors, and therapeutic helminth therapy have all been tried (Ribbons et al., 1997; Blackwood et al., 2008; Wilk et al., 2008; Broadhurst et al., 2012; Ferrecchia & Hobbs, 2013; Ardeshir et al., 2014). Tylosin was effective during the treatment period reported, but relapses occured for several patients after treatment cessation (Blackwood et al., 2008). Inulin and therapeutic helminth therapy showed some promise, but require further investigation (Broadhurst et al., 2012; Ardeshir et al., 2014).

The pathogenesis of ICE is not well understood. Perturbations in gastrointestinal microbial communities (dysbiosis) and altered immune system activity have been implicated (Sestak et al., 2003; McKenna et al., 2008; Wilk et al., 2008). Contemporary developments in the treatment of chronic colitis in humans have demonstrated the importance of the gastrointestinal microbiome in managing these conditions (Sartor, 2008; Mowat et al., 2011; Nahidi et al., 2013; Missaghi et al., 2014). Resident microbial communities prevent establishment of pathogenic organisms in the intestines and exert effects on the immune system directly (Hall et al., 2008; Ivanov et al., 2008; Sartor, 2008; Bordon, 2011; Geuking et al., 2011; Morgan et al., 2012). Intestinal microbial communities direct mucosal and systemic immune responses which may lead to an overall more balanced immune state and self-tolerance (Ivanov et al., 2008; Ochoa-Repáraz et al., 2009; Atarashi & Honda, 2011; Littman & Pamer, 2011).

Here we describe the first trial of a therapy for ICE using two antibiotics (vancomycin and neomycin) and an antifungal agent (fluconazole). We hypothesized that the microbial community present in animals with ICE contributed to acquiring or maintaining their disease, and that resetting gut community composition would improve health. Previously vancomycin and neomycin were used to reduce bacterial load in mice to allow introduction of new gut bacterial taxa (Shen et al., 2016). Currently the combination of vancomycin, neomycin and fluconazole is being tested for possible therapeutic effect in humans with inflammatory bowel diseases (L Albenberg and G Wu, pers. comm., 2014). The antifungal was included here because yeast overgrowth may occur after antibiotic treatment reduces gut microbial abundance (Kennedy & Volz, 1985; Samonis et al., 1993; Krause et al., 2001; Dollive et al., 2013; Schnorr et al., 2014). Antifungal agents have not previously been employed in the treatment of ICE in rhesus macaques. We found that the treatment was associated with a marked improvement in animal health, and followed up with a thorough study of the gut microbiota.

## Materials and Methods

### Subjects & housing

This study was conducted between June 2014 and July 2015. Housing and handling of animals were in accordance with the Animal Welfare Act and the Guide for the Care and Use of Laboratory Animals (US Public Health Service). TNPRC is an AAALACi-accredited institution. All protocols and procedures were reviewed and approved by the Tulane University Institutional Animal Care and Use Committee. Subjects included 18 (12 male, six female) juvenile (2.00–3.99 years old) Indian-origin rhesus macaques (*Macaca mulatta*) reared in outdoor breeding groups in the TNPRC specific pathogen free (SPF) breeding colony. The SPF breeding colony has been derived to exclude the following: Simian retrovirus Type D (SRV), simian T-lymphotrophic virus (STLV1), simian immunodeficiency virus (SIV), macacine herpesvirus 1 (MHV-1), and tuberculosis (*Mycobacterium tuberculosis*). SPF animals are sampled and monitored at least biannually.

Subjects were selected from animals admitted to the hospital for evaluation of gastrointestinal disease. Inclusion criteria included the following: 45 days of soft or liquid stool observed in a 90 day period, three negative fecal parasite evaluations within a five day period, and three negative fecal bacterial cultures within a five day period. Stool samples were collected into sterile feces tubes (Sarstedt, Nümbrecht, Germany) using the scoops provided in each individual feces tube. Samples were collected from each subject’s cage pan within approximately 2 h of the AM light cycle. After collection the samples were immediately placed into −80 °C storage until shipment. Fecal parasitological evaluation was performed via standard zinc sulfate flotation and fecal smear light microscopic analysis. Aerobic and anaerobic fecal microbiological culture was performed to detect the presence of common nonhuman primate enteric bacterial pathogens (*Shigella flexneri*, *Campylobacter jejuni*, *Yersinia enterocolitica*, *Salmonella* spp., *Escherichia coli*). Stool samples were plated on MacConkey agar (BBL, Cockeysville, MD, USA), Hektoen enter agar (BBL), Campy CVA agar (BBL), CIN Yersinia agar (BBL), and *Yersinia* enrichment broth (Oxoid, Ltd., Basingstoke, UK). *Balantidum coli*, trichomonads, and *Campylobacter coli* were considered to be opportunistic organisms. Macaques yielding positive test results for the above pathogens were excluded from the study.

Twelve animals met inclusion criteria and were determined to be suffering from idiopathic chronic enterocolitis (ICE). Of these subjects, six were randomly chosen for ICE treatment and six assigned to the untreated group. No subject received antibiotics or other medications during the 90-day pre-treatment observation period. In addition, six animals matched for age and viral status with no history of gastrointestinal disease, normal physical examinations, and normal complete blood counts and serum chemistries were identified and enrolled to serve as untreated, healthy controls. Over the course of the study, all subjects were housed in the same room in standard indoor nonhuman primate cages at TNPRC (Covington, LA, USA). In an attempt to maximize conspecific social contact without interfering with individual animal fecal observations, animals within the same experimental group were initially allowed full contact pair housing on an intermittent basis during the day (approximately 0800 to approximately 1500) and separated into single housing overnight during the 90 day observation periods. During the treatment and sample collection phases of this study, animals were singly housed. All cages were furnished with perches, foraging devices, and manipulable objects.

Macaques were fed Purina LabDiet 5000 commercial monkey biscuits (PMI Nutritional International, Brentwood, MO, USA) twice daily. Major ingredients (in order as per the manufacturer’s label) included dehulled soybean meal, ground corn, wheat middlings, ground soybean hulls, porcine animal fat preserved with BHA and citric acid, fish meal, sucrose, whole wheat, calcium carbonate, salt, ground oats, wheat germ and brewers dried yeast. Water was available ad-libitum, lighting was on a 12:12-h light:dark cycle, and temperature was maintained at 72 ± 5 °F (22.2 ± 1.2 °C).

### Data collection

Animals were observed daily by three trained and experienced staff for presence and character of feces as per standard husbandry practices at TNPRC. Staff were blinded to treatment group. Standard husbandry practice utilizes a simple 3-point scoring system for stool consistency. Feces were characterized as either normal, soft, or liquid based upon the appearance of the stool in the cage. Feces observed to be soft or liquid were classified as abnormal for the purposes of this study. In order to assess the ongoing health status of healthy controls and subjects with ICE, physical examinations were performed and blood was collected for complete blood counts and serum chemistry. Blood was collected via femoral venipuncture using standard venipuncture techniques in nonhuman primates. Physical examinations on each subject were performed at each access under ketamine HCl anesthesia (10 mg/kg, IM; Butler Animal Health, Dublin, OH, USA). Complete blood count (CBC) and serum chemistries (Chem) were performed on blood samples obtained at the time points listed below. Blood and stool from all groups were collected for analysis 24 h prior to a duodenoscopy/colonoscopy procedure during which 10–20 duodenal and colonic mucosal biopsy samples were collected via standard endoscopic methods described here. All animals were maintained under general anesthesia. For upper GI endoscopy, an appropriately-sized mouth gag was inserted between the upper and lower dental arcades to prevent damage to the flexible endoscope. An Olympus EVIS Exera GIF Type H180 (Olympus Corporation of the Americas, Center Valley, PA, USA) flexible gastrointestinal endoscope was passed through the mouth gag and advanced through the esophagus into the gastric lumen. The stomach was insufflated with air to facilitate visualization of the pylorus. The stomach was visually inspected for abnormalities, and the endoscope was advanced through the pylorus into the proximal duodenum. The endoscope was then advanced approximately 5–10 cm past the major duodenal papilla. Olympus FB-25K-1 round-cup biopsy forceps (Olympus Corporation of the Americas, Center Valley, PA, USA) were inserted through the biopsy channel of the endoscope and were used to obtain 10–20 mucosal biopsies. Tissue biopsies were immediately placed on ice and stored in −80 °C until shipment. For colonoscopy, no prior preparation was used. An Olympus EVIS Exera GIF Type H180 flexible gastrointestinal endoscope was lubricated with sterile lubricating jelly (Henry Schein, Inc, Melville, NY, USA) and introduced per rectum and advanced retrograde approximately 15–20 cm. Visualization was achieved via saline irrigation. Olympus FB-25K-1 round-cup biopsy forceps were inserted through the biopsy channel of the endoscope and were used to obtain 10–20 mucosal biopsies. Tissue biopsies were immediately placed on ice and stored in −80 °C until shipment. The same veterinarian performed all endoscopic sample collections for this project. Immediately following duodenoscopy/colonoscopy, the six affected animals chosen at random to serve as the ICE-treated group received the following oral medications: 125 mg total vancomycin hydrochloride (Hospira, Inc., Lake Forest, IL, USA) PO four times daily, 50 mg/kg neomycin (Bimeda, Inc., Le Suer, MN, USA) PO twice daily, and 2.5 mg/kg fluconazole (PACK Pharmaceuticals, LLC., Buffalo Grove, IL, USA) PO twice daily beginning on Day 0 and continuing for 14 days. This drug combination was chosen based on anecdotal reports of improvement observed in human cases of chronic gastrointestinal disease. (L Albenberg and G Wu, pers. comm., 2014). We chose to add fluconazole due to reports of yeast overgrowth in the GI tract during antibiotic therapy (Kennedy & Volz, 1985; Samonis et al., 1993). During pre-treatment and inter-treatment observation, no potentially confounding food enrichment was administered with the exception of orange halves through which the oral therapeutic agents were administered. The ICE-untreated and healthy control groups also received unadulterated orange halves on the same schedule as outlined for the ICE-treated group above. The following sample collection schedule applied to all three experimental groups (ICE-treated, ICE-untreated, and healthy controls). On Day 7 post treatment initiation, stool was collected from all animals for analysis. On Day 14 blood, stool, and mucosal biopsies were collected (as described above) from all animals. On Day 21 blood was collected from all participants. On Day 28 blood and stool were collected from all groups prior to a final endoscopy/colonoscopy with mucosal biopsy collection. Stool and mucosal biopsies were stored at −80 °C until shipped on dry ice for processing and sequencing. Additionally, monkey biscuits (Purina LabDiet 5000) were sequenced and analyzed as described below for the fecal and mucosal biopsy samples.

### DNA isolation and sequencing

DNA was isolated from stool, mucosal biopsies and food samples using the MoBio PowerSoil Kit (96-well format) in a sterile class II laminar flow hood. Prior to isolation, standard aliquots of sample were weighted and placed into a plate well with beads and buffer, and homogenized for 20 min on the TissueLyser II (Qiagen Valencia, CA, USA). DNA was isolated using the manufacturer’s protocol and then quantified using Picogreen. Amplicons were sequenced using the MiSeq 2 × 250 kit.

### Bacterial 16S rRNA gene quantification

Bacterial communities were assessed using the V1–V2 region of the bacterial 16S rRNA gene. The 16S gene was amplified using universal 16S primers 27F and 338R with Golay barcodes for each sample (see Table S1) (Liu et al., 2007). PCR amplifications were conducted in triplicate with AccuPrime Taq DNA Polymerase High Fidelity (ThermoFisher Scientific, Waltham, MA, USA). The reaction mixture, prepared in a PCR clean room, contained 7.21 uL PCR-grade H2O, 2.5 ul 10× buffer II, 0.19 ul Taq, 5 ul each forward and reverse primer (2 uM) and 5 uL DNA template.

The PCR was performed on an Applied Biosystems GeneAmp PCR System 9700 (ThermoFisher Scientific Inc., Waltham, MA, USA) using the following cycling conditions: initial denaturation at 95 °C for 5 min, 30 cycles of denaturation at 95 °C for 30 s, annealing at 56 °C for 30 s, and extension at 72 °C for 90 s, and then a final extension of 8 min at 72 °C. Replicate amplicons were pooled and bead purified using Agencourt AMPure XP (Beckman Coulter, Brea, CA, USA) using the manufacturer’s protocol. The amplicons were sequenced on an Illumina Miseq. Numbers of reads recovered are summarized in Figs. S1A and S1B.

### DNA sequencing of the fungal ITS rRNA locus

Fungal communities were assessed by sequencing the eukaryotic ITS1 rRNA locus. This region was amplified using ITS1F (CTTGGTCATTTAGAGGAAGTAA) and ITS2 (GCTGCGTTCTTCATCGATGC) primers specific for fungal ITS regions and Golay barcodes for each sample (see Table S1) (Gardes & Bruns, 1993; Charlson et al., 2012). The reaction mixture was comprised of 12.1 ul PCR-grade water, 2.5 ul Buffer II, 3 ul 10 uM each forward and reverse primer, 0.4 ul of AccuPrime Polymerase, and 4 ul of template. Each reaction was performed in triplicate with the following cycling conditions: initial denaturation at 94 °C for 3 min; 35 cycles of 94 °C for 45 s, then 56 °C for 60 s, then 72 °C for 90 s; and then a final extension at 72 °C for 10 min. The replicates were pooled and bead-purified using Agencourt AMPure XP beads at a 1.0 ratio, and then purified again with a 0.8 ratio to remove primer dimers. Amplification products were checked on a BioAnalyzer and quantified using Picogreen. The amplicons were pooled and a final bead purification at 0.8 ratio was performed to remove remnant primer dimers. The amplicons were sequenced on an Illumina Miseq.

### Bacterial community analysis

The 16S rRNA gene sequences were analyzed using the QIIME 1.91 pipeline; additional analysis was conducted in the R programming language. Quality filtering of reads was performed using standard QIIME parameters. Operational taxonomic units (OTUs) were created by clustering the quality-controlled reads at 97% identity using SWARM (Mahé et al., 2014). Representative sequences from each OTU were aligned using PyNAST (Caporaso et al., 2010). OTUs studied are compiled in Table S2. Lineages were attributed using the Greengenes database (McDonald et al., 2012).

### Fungal community analysis

ITS reads were processed using the PIPITS (Gweon et al., 2015) pipeline as follows: Reads were joined using PEAR and quality filtered using FAST-X toolkit. ITS1 regions in each read (if present) were identified and extracted using a hidden Markov model via ITSX (Pal et al., 2014). The ITS1 regions were then clustered at 97% identity using VSEARCH (Rognes et al., 2016). Representative sequences were then assigned a taxonomy using the RDP classifier and Warcup V1 ITS database (Deshpande et al., 2016). Subsequent analysis was performed in R. OTUs studied are compiled in Table S3.

### Sequence data access

All DNA sequences determined in this study are available at NCBI under PRJNA395230.

### Statistical analysis

For both bacterial and fungal communities in stool, indicator lineages were identified via the method of Dufrêne & Legendre (1997) and as implemented in the indicspecies R package (Dufrêne & Legendre, 1997; De Cáceres & Legendre, 2009). The abundances of the OTUs most strongly identified as indicator lineages were used as inputs to a random Forest classifier (R package randomForest, Liaw & Wiener, 2002), which was trained and tested using the default parameters for the package. The OTUs were ranked by importance by measuring the decrease in classification accuracy that resulted from removing each OTU from the classifier. For bacterial and fungal communities in colon biopsy samples, differentially abundant lineages were determined using a negative binomial model as implemented in the R package DESeq2 (Love, Huber & Anders, 2014) with an FDR-corrected *p*-value less than 0.05. For all differential abundance tests, the study groups were as follows: “Healthy” were samples belonging to monkeys in the Day 0 healthy, untreated group or Day 21 ICE-treated group (*n* = 12). “Unhealthy” samples were those belonging to monkeys in the Day 0 ICE-treated group or the Day 0 ICE-untreated group (*n* = 12).

Clinical effects of treatment were assessed by comparing affected and unaffected animals. For the treated subjects, the total proportion of days in which abnormal stool was observed was compared between a 90 day pre-treatment phase after admission to the hospital for diarrhea, and a 90 day post-treatment phase beginning immediately after the 14 day course of treatment. For untreated ICE-affected subjects, values were calculated over corresponding 90-day phases. A repeated measures ANOVA with phase as the within-subjects variable and treatment group as the between subjects variable was conducted with an alpha of 0.05 and a trend defined as *p* < 0.10. Where significant interactions were found, planned post-hoc comparisons were conducted with an alpha of 0.025 to adjust for multiple comparisons.

## Results

### Clinical effect

**Figure 1:**
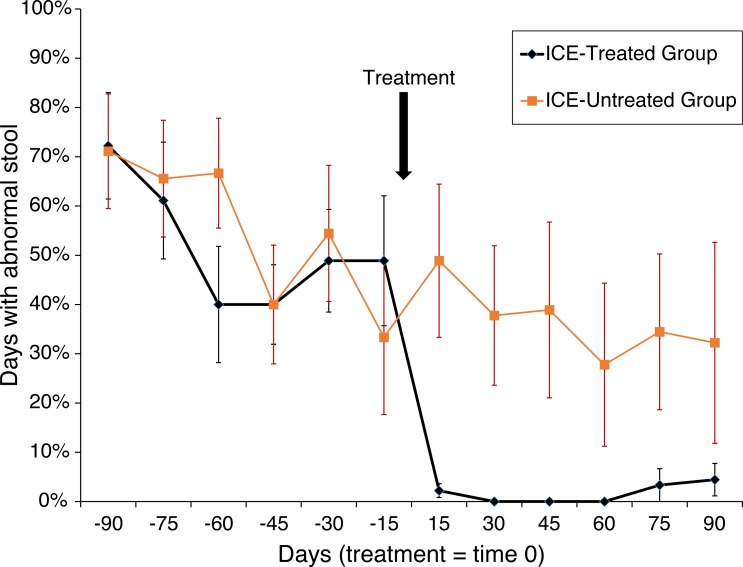
Treatment of animals with ICE results in reduced occurrence of abnormal stool. Reduction in abnormal stool frequency was observed with treatment. Animals were monitored over 180 days (*x*-axis) with the time of treatment plotted as time zero. On the *y*-axis is plotted the percent of days since the last time point with abnormal stool. Healthy control group data were not included as abnormal stool was observed in only one subject on one day during the observation period.

Animals were tested for abnormal stool composition (i.e., liquid, soft, or normal), and the effects of treatment were assessed (*n* = 6 in each of the three groups). An effect in the treatment group was found (*F*_(1,10)_ = 19.3; *p* < 0.001) with a trend towards interaction between phase (i.e., before or after treatment) and treatment (*F*_(1,10)_ = 4.04; *p* > 0.001). Among the treated subjects, days with abnormal stool fell from 51.9% to 1.7% (*p* < 0.0005) but remained unchanged in the untreated subjects (55.2% vs. 36.7%; N.S.; Fig. 1). A more dramatic pattern was found for liquid stool alone, the most severe manifestation of the disease, with a main effect of treatment group (*F*_(1,10)_ = 32.67; *p* < 0.0001) and an interaction between treatment group and phase (*F*_(1,10)_ = 12.62; *p* > 0.001). Whereas during the pre-treatment phase 36.5% of affected subjects showed liquid diarrhea, none was observed following treatment (*p* < 0.0004), and no change was detected among untreated subjects (31.0% vs. 22.2%; N.S.). Abnormal stool was observed on one day for one animal in the healthy untreated control group.

No pathogenic agents were identified via fecal parasitological analysis and microbiological culture of rectal swabs taken serially in each of the ICE-affected animals as part of their initial screening for inclusion. Serial complete blood count (CBC) and serum chemistry (Chem) results from samples taken throughout the observation, treatment, and sample collection periods did not differ from healthy control animals. These findings are consistent with cases of ICE observed at TNPRC and other facilities housing nonhuman primates (McKenna et al., 2008; Broadhurst et al., 2012; Ferrecchia & Hobbs, 2013; Kanthaswamy et al., 2014).

### Microbiome analysis of bacterial taxa

We sought to determine whether microbial community structure in macaque gut could be associated with ICE. Samples were collected from all animals in the three groups on specific treatment days in the ICE-treated group and on corresponding days in the untreated groups (ICE-treated and healthy untreated). Collection from treated and untreated animals occurred on Days 0, 14, and 21. Duodenal and colonic biopsies were also collected endoscopically.

**Figure 2:**
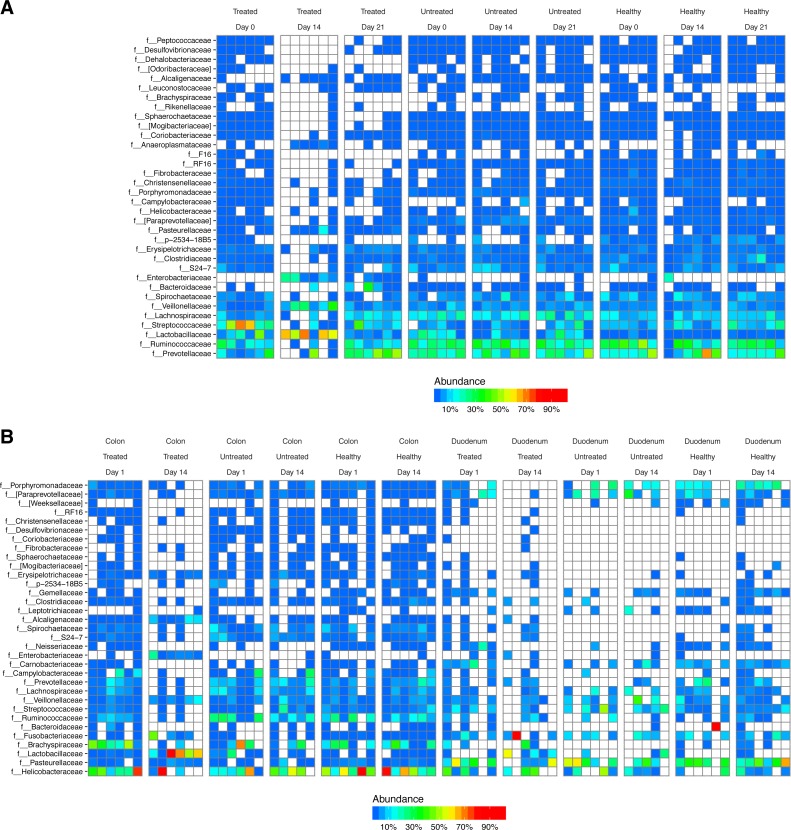
Bacterial lineages detected by 16S rRNA gene sequencing in healthy controls, and animals with ICE that did or did not receive the antibiotic/antifungal treatment. Columns indicate each time point sampled; rows indicate bacterial lineages. Abundances of the bacterial lineages detected is shown by the color scale. (A) Samples from stool. (B) Samples from gut tissue biopsies. A comparison of the stool samples to sequences from DNA in macaque biscuits is in Fig. S1.

After DNA extraction, PCR amplification with 16S rRNA gene primers and sequencing, we recovered an average of 16,484 reads from stool samples, 4,784 from colonic tissue, 246 from duodenum, and 73 from negative controls (Fig. S1). The major bacterial lineages detected are shown in Fig. 2. The most abundant lineages in stool included Prevotellaceae, Ruminococcaceae, Lactobacillaceae, and Streptococcaceae. Other lineages less commonly found in humans, such as Treponema (family Spirochaetaceae) and Bacteriodes S24-7, were also identified. Some macaques in the ICE-treated group showed higher levels of Streptococcaceae than macaques in the ICE-untreated group at baseline. During the period of antibiotic and antifungal treatment of the ICE-cohort, Lactobacillaceae predominated, which was a departure from the baseline composition. We infer that the Lactobacillaceae lineages detected were insensitive or less sensitive to the antibiotic treatment than other community members.

Biopsies yielded fewer sequences, and not all samples yielded useable data. The bacterial populations associated with the tissue sites were distinct from those in feces. Major bacterial lineages in the colon included Helicobacteraceae and Brachyspiraceae and Pasteurellaceae in the duodenum. Here, too, a notable feature was the presence of high Lactobacillaceae in colon biopsies during the period of antibiotic treatment (4/6 samples).

### Association of bacterial community structure and ICE

**Figure 3:**
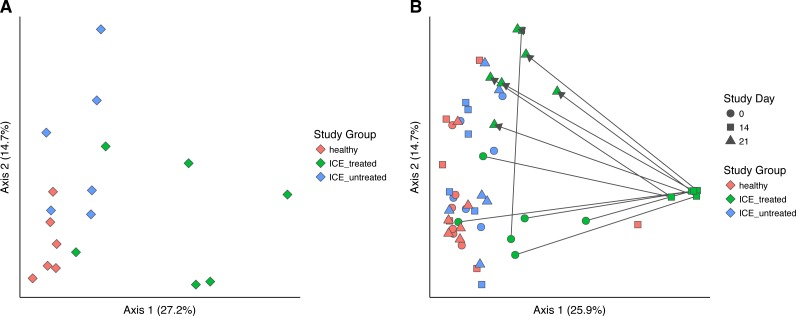
Generalized UniFrac distances were used to perform principal coordinate analysis (PCoA) of bacterial community structure. (A) PCoA showing only samples from Day 0 in all groups. A significant difference in community structure between study groups was found using PERMANOVA (*p* < 0.001). (B) Longitudinal analysis of bacterial community structure. Time is indicated with the symbol shape, and the study group by color. A significant difference between communities (excluding Day 14 samples) was found using PERMANOVA (*p* = 0.015).

Bacterial community structure was compared using generalized UniFrac, which measures a distance between pairs of communities by aligning all OTUs detected for each pair of samples onto a common phylogenetic tree and scores the shared branch length on the tree (Chen et al., 2012). Generalized UniFrac takes into account both presence/absence and the abundance of OTUs when calculating community distance. Comparison of the three communities at time zero showed a significant difference in community structure among the three groups (Fig. 3A, *p* < 0.001, PERMANOVA), leaving the influence of ICE in isolation unclear.

We next compared the treated and untreated stool samples over all time points (Fig. 3B). The most notable outliers were the samples collected at Day 14 from the ICE-treated group, associated with the antimicrobial treatment. As mentioned above, the Day 14 samples from the treated group were dominated by certain Lactobacillaceae which we infer are relatively less sensitive to the antibiotic regimen (Fig. S2). Apart from Day 14 treated samples, only a weak effect of ICE versus health was evident (*p* = 0.015, *R*2 = 0.1). We did not compare the tissue biopsies due to their lower sample numbers.

We also queried the association of stool consistency and community structure. No significant association was detected (Fig. S3).

### Bacterial lineages potentially associated with ICE

We sought to identify bacterial lineages selectively associated with clinical signs of ICE. For the analysis, we pooled healthy and unhealthy specimens. Specifically for healthy, we included untreated control monkeys on Day 1 and ICE-treated monkeys on Day 21 (absence of clinical signs of ICE) in both stool and intestinal biopsy samples. For the unhealthy (clinical signs of ICE) group, we included the ICE untreated group and the ICE-treated group (clinical signs of ICE) on Day 1. We excluded samples from Day 14 of the ICE treated group in favor of Day 21 in order to avoid allowing the effect of active treatment to dominate the classifier.

**Figure 4:**
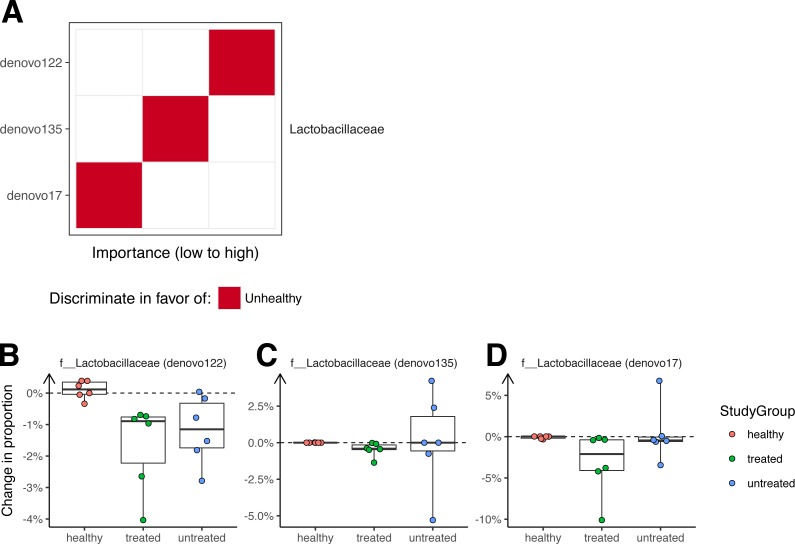
Bacterial lineages enriched in ICE cases. (A) Importance scores for notable lineages used in the Random Forest Model. A Random Forest Model was generated that discriminated healthy samples from ICE samples. For this, we pooled equal numbers of samples from healthy monkeys and monkeys 21 days after treatment as the “healthy” cohort, and samples from ICE afflicted, untreated monkeys as the “unhealthy” cohort. The importance of each lineage to the classifier error was calculated by measuring the change in out-of-bag error rate with that lineage removed. (B) Lactobacillaceae (denovo122) lineages in stool potentially associated with ICE. (C) Lactobacillaceae (denovo135) lineages in stool potentially associated with ICE. (D) Lactobacillaceae (denovo17) lineages in stool potentially associated with ICE. Each point corresponds to a separate animal. Colors indicate the study groups.

A Random Forest classifier was developed to sort unhealthy and healthy monkey samples (Fig. 4). Testing of the classifier showed that it could classify the animals into their original groups (no clinical signs or clinical signs of ICE) with 95% accuracy (5% out of bag error rate). As a future control for over fitting, it will be useful to determine whether this model can also distinguish ICE cases from healthy controls in new data sets not used to generate the model.

Assessment of the lineages primarily responsible for discrimination between healthy and unhealthy animals in colonic biopsy samples (Fig. 4A) showed that OTUs of the families Lactobacillaceae and Ruminococcaceae were the most prominently enriched in ICE cases, and are therefore candidates for ICE-associated organisms.

A separate analysis, using the same approach but with only time zero samples, showed that healthy animals were distinguished by the presence of *Christensenella* (Fig. S4) when compared to macaques with ICE. In these samples, two OTUs (denovo326 and denovo557), annotated as belonging to the Christensenellaceae family, were present at higher levels in the healthy cohort, and were important features for distinguishing between cohorts for the Random Forest classifier.

If the cause of ICE is due to the presence of a pathogenic bacteria, we reasoned that any causal organism should decrease in abundance with treatment, and so investigated changes in colonization over the treatment period (Fig. 4B). All of the OTUs in stool contributing to discrimination changed only slightly after treatment, with none showing a greater than 10% absolute change in abundance.

Discriminative lineages identified in stool were then compared to lineages in intestinal biopsies. Sparse bacterial detections and limited time points precluded generating an effective Random Forest classifier, and further analysis using a negative binomial model failed to identify discriminating taxa. Thus no signal of ICE could be discerned in the biopsy samples.

### Fungal taxa are derived primarily from food

**Figure 5:**
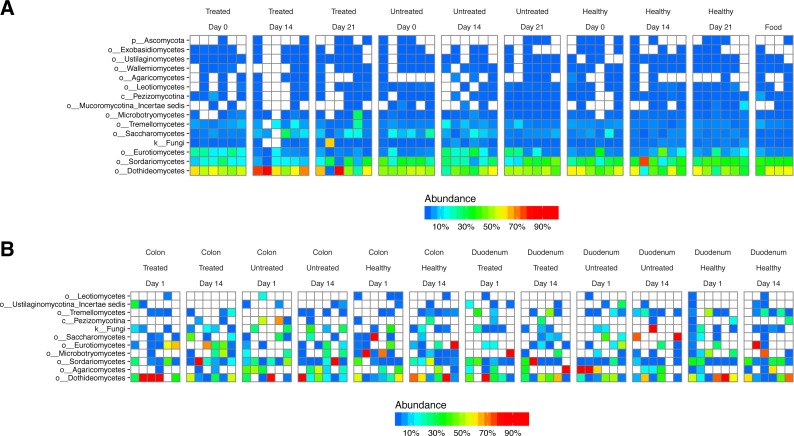
Fungal lineages detected using ITS ribosomal gene tag sequencing. Columns indicate samples studied, rows indicate fungal lineages. The color code quantifies abundance. (A) ITS sequences from fecal samples and food. (B) ITS sequences from gut tissue samples.

Because the successful therapy included an antifungal agent, we investigated fungal lineages for association with ICE. DNA samples from stool were amplified with ITS primers, which are selective for sequences from the eukaryotic rRNA locus. A variety of fungal lineages were detected in the samples (Fig. 5)—relatively abundant taxa in stool included Dothidiomycetes, Sordariomycetes, Eurotiomycetes, and Saccharomycetes. Analysis using PERMANOVA on UniFrac distances between samples showed no significant difference between ICE and control samples (*p* = 1). Lineages detected in tissue samples were sparse (Fig. 5B), and again no discrimination could be obtained using PERMANOVA. A random forest classifier was constructed as above with the goal of discriminating ICE from healthy animals, but no strongly discriminating taxa were identified.

We next investigated the degree to which lineages detected in our study originated as transients in food. We extracted DNA from macaque biscuits (their major food material) and carried out 16S and ITS rRNA tag sequencing. We found little resemblance between bacterial lineages from stool or biopsy samples and biscuit samples (Fig. S2A). However, fungal lineages were nearly identical in stool and biscuits samples (Fig. 5A). Labeling on the biscuit packaging revealed that brewers dried yeast was a component of the biscuits. We thus conclude that the fungal sequences detected in stool corresponded primarily to transients or free DNA from food, whereas the bacterial sequences were mostly from gut-resident organisms.

## Discussion

This study represents the first trial of an oral combination therapy comprised of vancomycin, neomycin, and fluconazole as a treatment for ICE in juvenile rhesus macaques. The treatment was derived from regimens used to prepare mice for human microbiota transplants and is currently being tested for therapeutic effect in human IBD (Dollive et al., 2013; Hintze et al., 2014). All animals treated displayed improvement in stool consistency, which persisted over the post-treatment 90 day observation period. This therapeutic combination thus provides an attractive option for the management of ICE and potentially a tool for elucidating the etiologies of gastrointestinal disease.

Of the previously tested ICE treatment interventions, only tylosin, inulin, and therapeutic helminth therapy were able to demonstrate clear clinical improvements (Blackwood et al., 2008; Broadhurst et al., 2012; Ardeshir et al., 2014). Tylosin treatment yielded significant clinical improvements; however, relapses were noted in 39% of animals within 30 days after treatment cessation (Blackwood et al., 2008). These results are consistent with a similar disease of dogs (*Canis lupus familiaris*) called tylosin-responsive or antibiotic-responsive diarrhea (Westermarck et al., 2005; Kilpinen, Spillmann & Westermarck, 2014; Kilpinen et al., 2015). Therapeutic helminth therapy provided similar long-term clinical improvements; however, the authors recommend further studies including a sham-treated control arm in order to control for spontaneous remission (Broadhurst et al., 2012).

Bacterial lineages belonging to the Christensenellaceae family were found to be important differentiators between healthy and ICE-affected macaques prior to treatment. *Christensenella* has been shown to be a heritable, health-associated gut microbe in humans (Goodrich et al., 2014). The enrichment of *Christensenella* has been associated with lower body mass index and protection from obesity in humans. It is possible that in macaques *Christensenella* bacteria play a similarly beneficial role and their absence in ICE reflects a dysbiotic state.

The major fungal lineages detected, unexpectedly, could be traced to food additives. Bacterial lineages, in contrast, did not match between stool and food, emphasizing that the large gut-resident bacterial communities predominated. Evidently macaque gut fungal communities are sufficiently sparse that food sequences predominate. It is unclear whether the fungal sequences detected derived from fungal cells, or fungal DNA remaining in processed macaque biscuits. Previous studies have shown that DNA in food can pass through the mouse gut and remain sufficiently intact for detection in mouse feces (Dollive et al., 2013).

This study has several limitations. A major limitation is the small sample size. Temporal changes in fecal consistency scores were observed during this study and others, emphasizing the possible role of changes unrelated to therapy (Ferrecchia & Hobbs, 2013). This waxing and waning of clinical signs in ICE is consistent with chronic enterocolitis in several other species (Kalck, 2009; Jergens & Simpson, 2012). Additional study is needed in further cohorts to establish that our results are a consequence of the treatment applied. Future studies should also evaluate the duration of clinical response beyond the 90 day observation period used here. Other limitations include a wider age range and higher median age in the healthy control group and the use of sequence tags to assess microbial taxa. Regarding the use of sequence tags, all tag systems will recover some but not all microbial lineages, and the ITS tag used are known to have gaps in the fungal taxa queried (Dollive et al., 2012; Dollive et al., 2013). The difference in ages between the ICE-treated, ICE-untreated, and healthy control groups presents a potential confounding factor with regard to the differences observed in the bacterial communities, so these differences should be interpreted cautiously (Fig. S7). While all animals enrolled in this study are considered juveniles, the sexual maturation process in macaques is extremely variable with regard to age of onset and duration (Wilson et al., 1984; Luetjens & Weinbauer, 2012). Previous studies have shown that hormonal changes which occur during sexual maturation in humans have demonstrable effects on the gastrointestinal microbiome (Agans et al., 2011).

We expected that antimicrobial therapy would be correlated with measureable changes in the microbiome, but observed changes were slight. The largest changes were associated with antibiotic treatment rather than ICE versus health. Our data suggest that elevated levels of certain Lactobacillaceae may be associated with ICE, but these bacteria were relatively low in abundance and showed only modest changes associated with therapy. Furthermore, removing the two Lactobacillaceae OTUs (denovo122 and denovo135) from the Random Forest classifier had a limited effect on classification accuracy (5–8% increase in error rate), suggesting that they are not the sole discriminant between ICE and health. These data thus provide hypotheses for future studies but no clear etiological agent for ICE. It seems more likely that ICE is caused by perturbations in community structure and loss of protective bacteria.

While the therapy assessed in this study demonstrated clinical improvement, caution and a thorough risk assessment is advised whenever an antibiotic therapy is prescribed. The mounting crisis of increasing antibiotic resistance makes it imperative that ethical and judicious use of antibiotics be instituted (Teuber, 2001; Ungemach, Müller-Bahrdt & Abraham, 2006). Though this treatment may not represent a viable overall clinical management strategy for all ICE-affected macaques within a colony, it offers an attractive option for managing this disease in specific cases involving high-value animals that would otherwise meet humane endpoints as a result of the ICE disease state.

## Conclusions

In summary, our study indicates that an oral therapeutic regimen of vancomycin, neomycin, and fluconazole effectively improves stool consistency in juvenile rhesus macaques affected by ICE. Our microbial analyses did not reveal any significant effects of this treatment on specific bacterial or fungal lineages present in the rhesus duodenal, colonic or fecal microbiota, generating potential hypotheses for further study on this important disease of captive nonhuman primates.

##  Supplemental Information

10.7717/peerj.4612/supp-1 Figure S1 Sequence reads acquired in this study The sequencing reads obtained for 16S (A) and ITS (B) are shown for each sample type. The y-axis is on a log scale.

10.7717/peerj.4612/supp-2 Figure S2 Heat map comparing 16S rRNA gene sequence data for stool to data negative controls and DNA extracted from macaque biscuits Alternative views of the bacterial lineages found in feces and tissue. In (A) the proportional abundance of each taxa in feces is shown grouped by study day and disease state. In (B) the abundance of each major taxa in tissue is shown alongside their proportion in the negative controls (blanks).

10.7717/peerj.4612/supp-3 Figure S3 PCoA of all stool samples, colored by stool consistency Principal coordinate decomposition (PCoA) plot showing breakdown of stool samples by consistency (color) and study day (shape).

10.7717/peerj.4612/supp-4 Figure S4 Proportion of reads in each sample ascribed to the two disease-state discriminating OTUs in ICE stool samples The taxonomic assignment for each OTU is shown above the plot.

10.7717/peerj.4612/supp-5 Figure S5 Heat map comparing ITS rRNA gene sequence data to negative controls and DNA extracted from macaque biscuits Alternative view of the fungal lineages shown in feces and tissue. In (A) the proportional abundance of each fungal taxa in feces is shown grouped first by study day and then by disease state. In (B) the major fungal taxa are shown in tissue samples alongside their proportion in the negative controls (blanks).

10.7717/peerj.4612/supp-6 Figure S6 Shannon diversity of the bacterial communities in each sample type (columns) and study day (rows) Each panel shows the bacterial diversity, measured by the Shannon index, of the three disease states (*x*-axis). The panels are separated by sample type (columns) and study day (rows). Each pairwise comparison was performed using the Wilcoxon rank-sum test and adjusted for multiple testing using FDR. *, *p* < 0.05; **, *p* < 0.01; NS, not significant.

10.7717/peerj.4612/supp-7 Figure S7 Age by group box plots Box plots representing age (in years) distribution between groups.

10.7717/peerj.4612/supp-8 Figure S8 Side-by-side bar graph depicting mean animal weights by group and their associated changes over time “Pre-treatment” values represent the mean of all weights during the 90-day pre-treatment observation period by group. “Treatment” values represent the mean of all weights during the 14-day treatment period by group. “Post-treatment” values represent the mean of all weights available during the 90-day post-treatment observation period by group.

10.7717/peerj.4612/supp-9 Table S1 DNA oligonucleotides used in this study

10.7717/peerj.4612/supp-10 Table S2 Bacterial OTUs generated from 16S rRNA gene sequence data

10.7717/peerj.4612/supp-11 Table S3 Fungal OTUs generated from ITS rRNA gene sequence data

10.7717/peerj.4612/supp-12 Supplemental Information 1 Full observation data for study subjects Subjects were observed daily for presence and character of stool. **N, Normal stool; S, Soft stool; L, Liquid stool.**

## Additional Information and Declarations

### Data Availability

The following information was supplied regarding data availability: Bioproject PRJNA395230. GenBank SRA accession number SRP115422.

## References

[ref-1] Agans R, Rigsbee L, Kenche H, Michail S, Khamis HJ, Paliy O (2011). Distal gut microbiota of adolescent children is different from that of adults. FEMS Microbiology Ecology.

[ref-2] Ardeshir A, Oslund KL, Ventimiglia F, Yee J, Lerche NW, Hyde DM (2013). Idiopathic microscopic colitis of rhesus macaques: quantitative assessment of colonic mucosa. Anatomical Record.

[ref-3] Ardeshir A, Sankaran S, Oslund K, Hartigan-O’Connor D, Lerche N, Hyde D, Dandekar S (2014). Inulin treatment leads to changes in intestinal microbiota and resolution of idiopathic chronic diarrhea in rhesus macaques. Annals of the American Thoracic Society.

[ref-4] Atarashi K, Honda K (2011). Microbiota in autoimmunity and tolerance. Current Opinion in Immunology.

[ref-5] Blackwood RS, Tarara RP, Christe KL, Spinner A, Lerche NW (2008). Effects of the macrolide drug tylosin on chronic diarrhea in rhesus macaques (*Macaca mulatto*). Comparative Medicine.

[ref-6] Bordon Y (2011). Mucosal immunology: colonic creatures are TReg teachers. Nature Reviews Immunology.

[ref-7] Broadhurst MJ, Ardeshir A, Kanwar B, Mirpuri J, Gundra UM, Leung JM, Wiens KE, Vujkovic-Cvijin I, Kim CC, Yarovinsky F, Lerche NW, McCune JM, Loke P (2012). Therapeutic helminth infection of macaques with idiopathic chronic diarrhea alters the inflammatory signature and mucosal microbiota of the colon. PLOS Pathogens.

[ref-8] Caporaso JG, Bittinger K, Bushman FD, Desantis TZ, Andersen GL, Knight R (2010). PyNAST: a flexible tool for aligning sequences to a template alignment. Bioinformatics.

[ref-9] Charlson ES, Diamond JM, Bittinger K, Fitzgerald AS, Yadav A, Haas AR, Bushman FD, Collman RG (2012). Lung-enriched organisms and aberrant bacterial and fungal respiratory microbiota after lung transplant. American Journal of Respiratory and Critical Care Medicine.

[ref-10] Chen J, Bittinger K, Charlson ES, Hoffmann C, Lewis J, Wu GD, Collman RG, Bushman FD, Li H (2012). Associating microbiome composition with environmental covariates using generalized UniFrac distances. Bioinformatics.

[ref-11] Damman CJ, Miller SI, Surawicz CM, Zisman TL (2012). The microbiome and inflammatory bowel disease: is there a therapeutic role for fecal microbiota transplantation?. American Journal of Gastroenterology.

[ref-12] De Cáceres M, Legendre P (2009). Associations between species and groups of sites: indices and statistical inference. Ecology.

[ref-13] Deshpande V, Wang Q, Greenfield P, Charleston M, Porras-Alfaro A, Kuske CR, Cole JR, Midgley DJ, Tran-Dinh N (2016). Fungal identification using a Bayesian classifier and the Warcup training set of internal transcribed spacer sequences. Mycologia.

[ref-14] Dollive S, Chen Y-Y, Grunberg S, Bittinger K, Hoffmann C, Vandivier L, Cuff C, Lewis JD, Wu GD, Bushman FD (2013). Fungi of the murine gut: episodic variation and proliferation during antibiotic treatment. PLOS ONE.

[ref-15] Dollive S, Peterfreund GL, Sherrill-Mix S, Bittinger K, Sinha R, Hoffmann C, Nabel CS, Hill DA, Artis D, Bachman MA, Custers-Allen R, Grunberg S, Wu GD, Lewis JD, Bushman FD (2012). A tool kit for quantifying eukaryotic rRNA gene sequences from human microbiome samples. Genome Biology.

[ref-16] Dufrêne M, Legendre P (1997). Species assemblages and indicator species: the need for a flexible asymmetrical approach. Ecological Monographs.

[ref-17] Ferrecchia CE, Hobbs TR (2013). Efficacy of oral fecal bacteriotherapy in rhesus macaques (*Macaca mulatta*) with chronic diarrhea. Comparative Medicine.

[ref-18] Gardes M, Bruns TD (1993). ITS primers with enhanced specificity for basidiomycetes—application to the identification of mycorrhizae and rusts. Molecular Ecology.

[ref-19] Geuking MB, Cahenzli J, Lawson MAE, Ng DCK, Slack E, Hapfelmeier S, McCoy KD, Macpherson AJ (2011). Intestinal bacterial colonization induces mutualistic regulatory T cell responses. Immunity.

[ref-20] Goodrich JK, Waters JL, Poole AC, Sutter JL, Koren O, Blekhman R, Beaumont M, Van Treuren W, Knight R, Bell JT, Spector TD, Clark AG, Ley RE (2014). Human genetics shape the gut microbiome. Cell.

[ref-21] Gweon HS, Oliver A, Taylor J, Booth T, Gibbs M, Read DS, Griffiths RI, Schonrogge K (2015). PIPITS: an automated pipeline for analyses of fungal internal transcribed spacer sequences from the Illumina sequencing platform. Methods in Ecology and Evolution.

[ref-22] Hall JA, Bouladoux N, Sun CM, Wohlfert EA, Blank RB, Zhu Q, Grigg ME, Berzofsky JA, Belkaid Y (2008). Commensal DNA limits regulatory T cell conversion and is a natural adjuvant of intestinal immune responses. Immunity.

[ref-23] Hintze KJ, Cox JE, Rompato G, Benninghoff AD, Ward RE, Broadbent J, Lefevre M (2014). Broad scope method for creating humanized animal models for animal health and disease research through antibiotic treatment and human fecal transfer. Gut Microbes.

[ref-24] Ivanov II, Frutos Rde L, Manel N, Yoshinaga K, Rifkin DB, Sartor RB, Finlay BB, Littman DR (2008). Specific microbiota direct the differentiation of IL-17-producing T-helper cells in the mucosa of the small intestine. Cell Host Microbe.

[ref-25] Jergens AE, Simpson KW (2012). Inflammatory bowel disease in veterinary medicine. Frontiers in Bioscience.

[ref-26] Kalck KA (2009). Inflammatory bowel disease in horses. Veterinary Clinics of North America: Equine Practice.

[ref-27] Kanthaswamy S, Elfenbein HA, Ardeshir A, Ng J, Hyde D, Smith DG, Lerche N (2014). Familial aggregation of chronic diarrhea disease (CDD) in rhesus macaques (*Macaca mulatta*). American Journal of Primatology.

[ref-28] Kennedy MJ, Volz PA (1985). Ecology of Candida albicans gut colonization: inhibition of Candida adhesion, colonization, and dissemination from the gastrointestinal tract by bacterial antagonism. Infection and Immunity.

[ref-29] Kilpinen S, Rantala M, Spillmann T, Björkroth J, Westermarck E (2015). Oral tylosin administration is associated with an increase of faecal enterococci and lactic acid bacteria in dogs with tylosin-responsive diarrhoea. The Veterinary Journal.

[ref-30] Kilpinen S, Spillmann T, Westermarck E (2014). Efficacy of two low-dose oral tylosin regimens in controlling the relapse of diarrhea in dogs with tylosin-responsive diarrhea: a prospective, single-blinded, two-arm parallel, clinical field trial. Acta Veterinaria Scandinavica.

[ref-31] Krause R, Schwab E, Bachhiesl D, Daxböck F, Wenisch C, Krejs GJ, Reisinger EC (2001). Role of Candida in antibiotic-associated diarrhea. Journal of Infectious Diseases.

[ref-32] Liaw A, Wiener M (2002). Classification and regression by randomForest. R News.

[ref-33] Littman DR, Pamer EG (2011). Role of the commensal microbiota in normal and pathogenic host immune responses. Cell Host Microbe.

[ref-34] Liu Z, Lozupone C, Hamady M, Bushman FD, Knight R (2007). Short pyrosequencing reads suffice for accurate microbial community analysis. Nucleic Acids Research.

[ref-35] Love MI, Huber W, Anders S (2014). Moderated estimation of fold change and dispersion for RNA-seq data with DESeq2. Genome Biology.

[ref-36] Luetjens CM, Weinbauer GF (2012). Functional assessment of sexual maturity in male macaques (*Macaca fascicularis*). Regulatory Toxicology and Pharmacology.

[ref-37] Mahé F, Rognes T, Quince C, De Vargas C, Dunthorn M (2014). Swarm: robust and fast clustering method for amplicon-based studies. PeerJ.

[ref-38] McDonald D, Price MN, Goodrich J, Nawrocki EP, Desantis TZ, Probst A, Andersen GL, Knight R, Hugenholtz P (2012). An improved Greengenes taxonomy with explicit ranks for ecological and evolutionary analyses of bacteria and archaea. ISME Journal.

[ref-39] McKenna P, Hoffmann C, Minkah N, Aye PP, Lackner A, Liu Z, Lozupone CA, Hamady M, Knight R, Bushman FD (2008). The macaque gut microbiome in health, lentiviral infection, and chronic enterocolitis. PLOS Pathogens.

[ref-40] Missaghi B, Barkema H, Madsen K, Ghosh S (2014). Perturbation of the human microbiome as a contributor to inflammatory bowel disease. Pathogens.

[ref-41] Morgan XC, Tickle TL, Sokol H, Gevers D, Devaney KL, Ward DV, Reyes JA, Shah SA, LeLeiko N, Snapper SB, Bousvaros A, Korzenik J, Sands BE, Xavier RJ, Huttenhower C (2012). Dysfunction of the intestinal microbiome in inflammatory bowel disease and treatment. Genome Biology.

[ref-42] Mowat C, Cole A, Windsor A, Ahmad T, Arnott I, Driscoll R, Mitton S, Orchard T, Rutter M, Younge L, Lees C, Ho GT, Satsangi J, Bloom S (2011). Guidelines for the management of inflammatory bowel disease in adults. Gut.

[ref-43] Nahidi L, Leach ST, Mitchell HM, Kaakoush NO, Lemberg DA, Munday JS, Huinao K, Day AS (2013). Inflammatory bowel disease therapies and gut function in a colitis mouse model. BioMed Research International.

[ref-44] Ochoa-Repáraz J, Mielcarz DW, Ditrio LE, Burroughs AR, Foureau DM, Haque-Begum S, Kasper LH (2009). Role of gut commensal microflora in the development of experimental autoimmune encephalomyelitis. Journal of Immunology.

[ref-45] Pal C, Bengtsson-Palme J, Rensing C, Kristiansson E, Larsson DGJ (2014). BacMet: antibacterial biocide and metal resistance genes database. Nucleic Acids Research.

[ref-46] Prongay K, Park B, Murphy SJ (2013). Risk factor analysis may provide clues to diarrhea prevention in outdoor-housed rhesus macaques (*Macaca mulatta*). American Journal of Primatology.

[ref-47] Ribbons KA, Currie MG, Connor JR, Manning PT, Allen PC, Didier P, Ratterree MS, Clark DA, Miller MJS (1997). The effect of inhibitors of inducible nitric oxide synthase on chronic colitis in the rhesus monkey. Journal of Pharmacology and Experimental Therapeutics.

[ref-48] Rice KA, Chen ES, Pate KAM, Hutchinson EK, Adams RJ (2013). Diagnosis of amyloidosis and differentiation from chronic, idiopathic enterocolitis in rhesus (*macaca mulatta*) and pig-tailed (M. nemestrina) macaques. Comparative Medicine.

[ref-49] Rognes T, Flouri T, Nichols B, Quince C, Mahé F (2016). VSEARCH: a versatile open source tool for metagenomics. PeerJ Preprints.

[ref-50] Samonis G, Gikas A, Anaissie EJ, Vrenzos G, Maraki S, Tselentis Y, Bodey GP (1993). Prospective evaluation of effects of broad-spectrum antibiotics on gastrointestinal yeast colonization of humans. Antimicrobial Agents and Chemotherapy.

[ref-51] Sartor RB (2008). Microbial influences in inflammatory bowel diseases. Gastroenterology.

[ref-52] Schnorr SL, Candela M, Rampelli S, Centanni M, Consolandi C, Basaglia G, Turroni S, Biagi E, Peano C, Severgnini M, Fiori J, Gotti R, De Bellis G, Luiselli D, Brigidi P, Mabulla A, Marlowe F, Henry AG, Crittenden AN (2014). Gut microbiome of the Hadza hunter-gatherers. Nature Communications.

[ref-53] Sestak K, Merritt CK, Borda J, Saylor E, Schwamberger SR, Cogswell F, Didier ES, Didier PJ, Plauche G, Bohm RP, Aye PP, Alexa P, Ward RL, Lackner AA (2003). Infectious agent and immune response characteristics of chronic enterocolitis in captive rhesus macaques. Infection and Immunity.

[ref-54] Shen TCD, Chehoud C, Ni J, Hsu E, Chen YY, Bailey A, Laughlin A, Bittinger K, Bushman FD, Wu GDW (2016). Dietary regulation of the gut microbiota engineered by a minimal defined bacterial consortium. PLOS ONE.

[ref-55] Snyder-Mackler N, Sanz J, Kohn JN, Brinkworth JF, Morrow S, Shaver AO, Grenier JC, Pique-Regi R, Johnson ZP, Wilson ME, Barreiro LB, Tung J (2016). Social status alters immune regulation and response to infection in macaques. Science.

[ref-56] Teuber M (2001). Veterinary use and antibiotic resistance. Current Opinion in Microbiology.

[ref-57] Ungemach FR, Müller-Bahrdt D, Abraham G (2006). Guidelines for prudent use of antimicrobials and their implications on antibiotic usage in veterinary medicine. International Journal of Medical Microbiology.

[ref-58] Westermarck E, Skrzypczak T, Harmoinen J, Steiner JM, Ruaux CG, Williams DA, Eerola E, Sundbäck P, Rinkinen M (2005). Tylosin-responsive chronic diarrhea in dogs. Journal of Veterinary Internal Medicine.

[ref-59] Wilk JL, Maginnis GM, Coleman K, Lewis A, Ogden B (2008). Evaluation of the use of coconut to treat chronic diarrhea in rhesus macaques (*Macaca mulatta*). Journal of Medical Primatology.

[ref-60] Wilson ME, Gordon TP, Blank MS, Collins DC (1984). Timing of sexual maturity in female rhesus monkeys (*Macaca mulatta*) housed outdoors. Journal of Reproduction and Fertility.

