# Supplementary figures and images for: Evaluation of a therapy for Idiopathic Chronic Enterocolitis in rhesus macaques (Macaca mulatta) and linked microbial community correlates
Source: PeerJ. 2018 Apr 11;6:e4612. doi: 10.7717/peerj.4612 (PMC5899420; doi:10.7717/peerj.4612)

**A** 16S reads per sample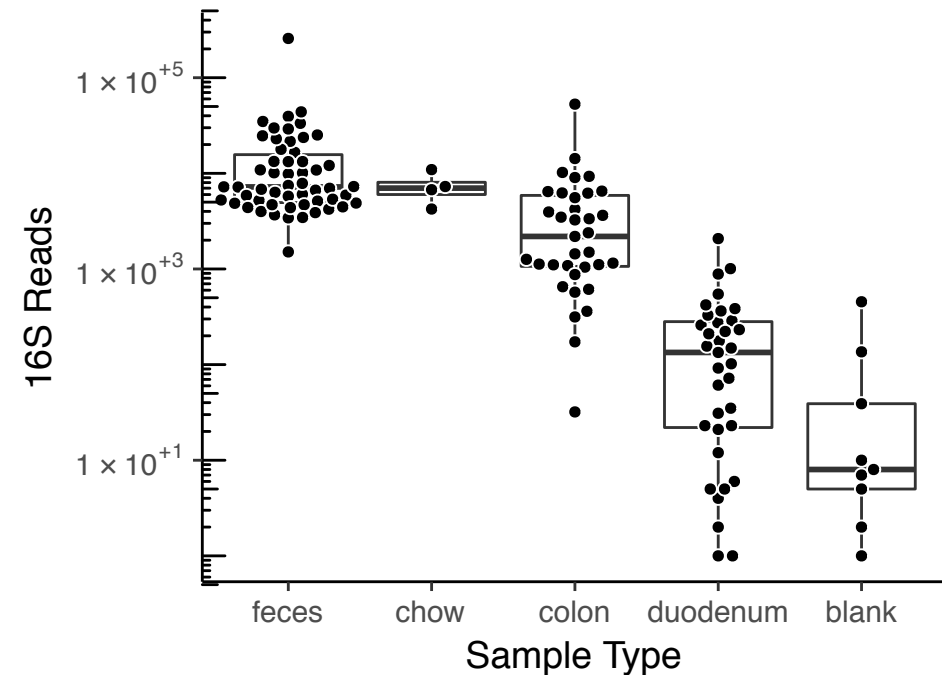**B** ITS reads per sample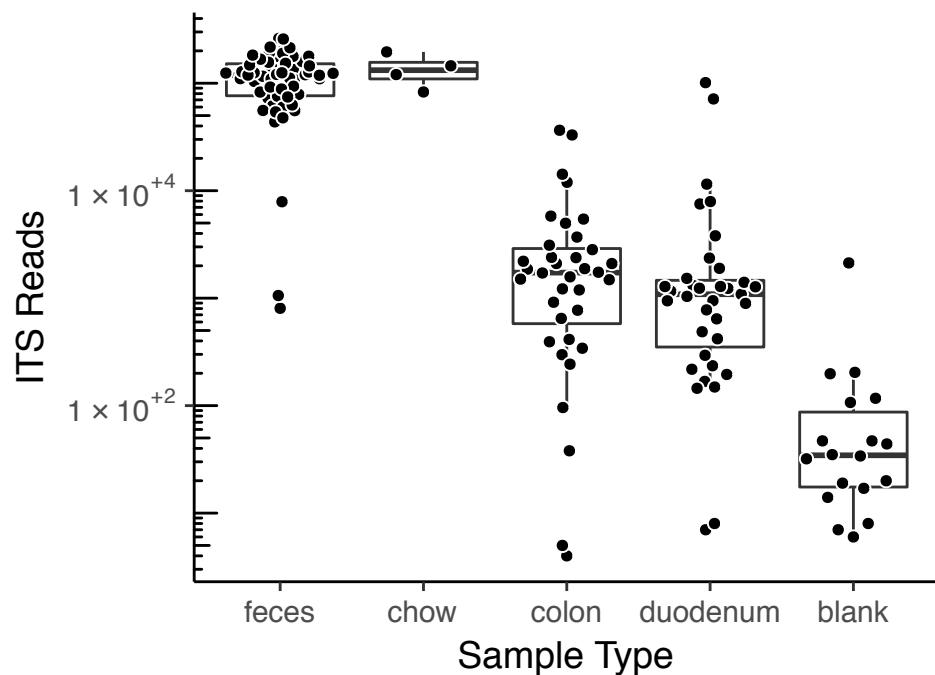

Supplement: Figure S1 — The sequencing reads obtained for 16S (A) and ITS (B) are shown for each sample type. The y-axis is on a log scale. [file peerj-06-4612-s001.pdf]

A

Bacterial taxa in feces; proportions

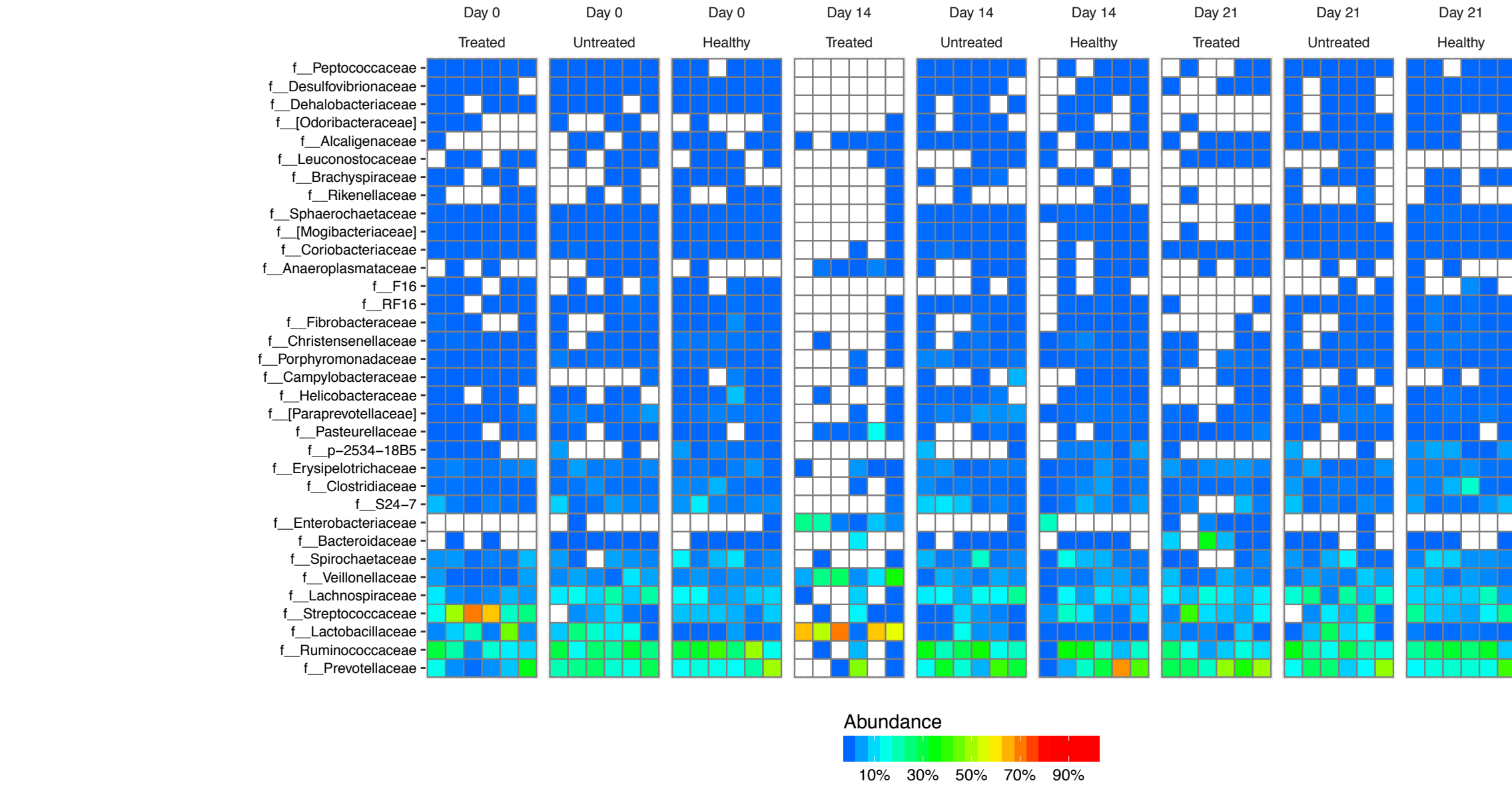

B

Bacterial taxa in tissue; raw reads, blanks

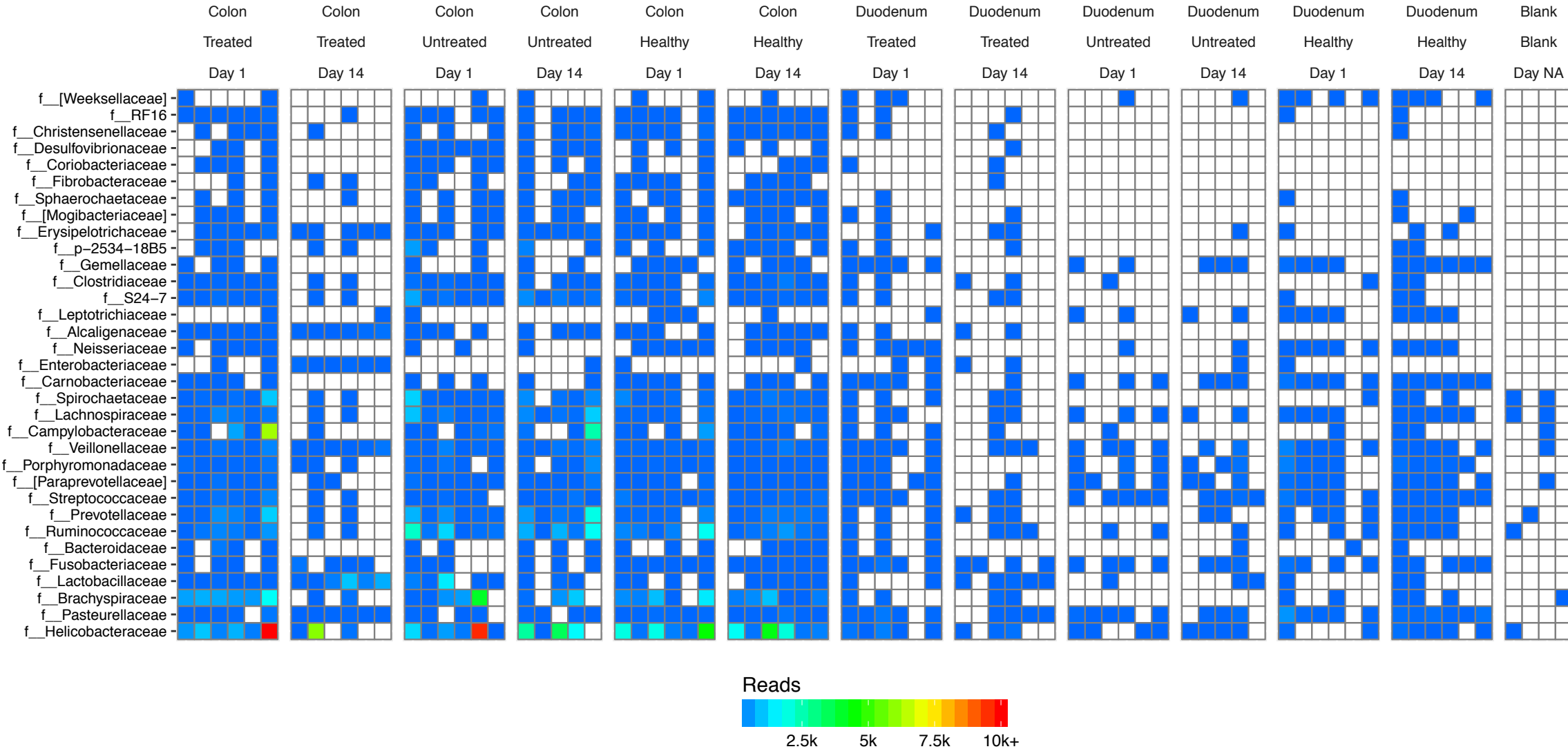

Supplement: Figure S2 — Alternative views of the bacterial lineages found in feces and tissue. In (A) the proportional abundance of each taxa in feces is shown grouped by study day and disease state. In (B) the abundance of each major taxa in tissue is shown alongside their proportion in the negative controls (blanks). [file peerj-06-4612-s002.pdf]

# Generalized UniFrac PCoA, 16S, feces only

Alpha = 0.5

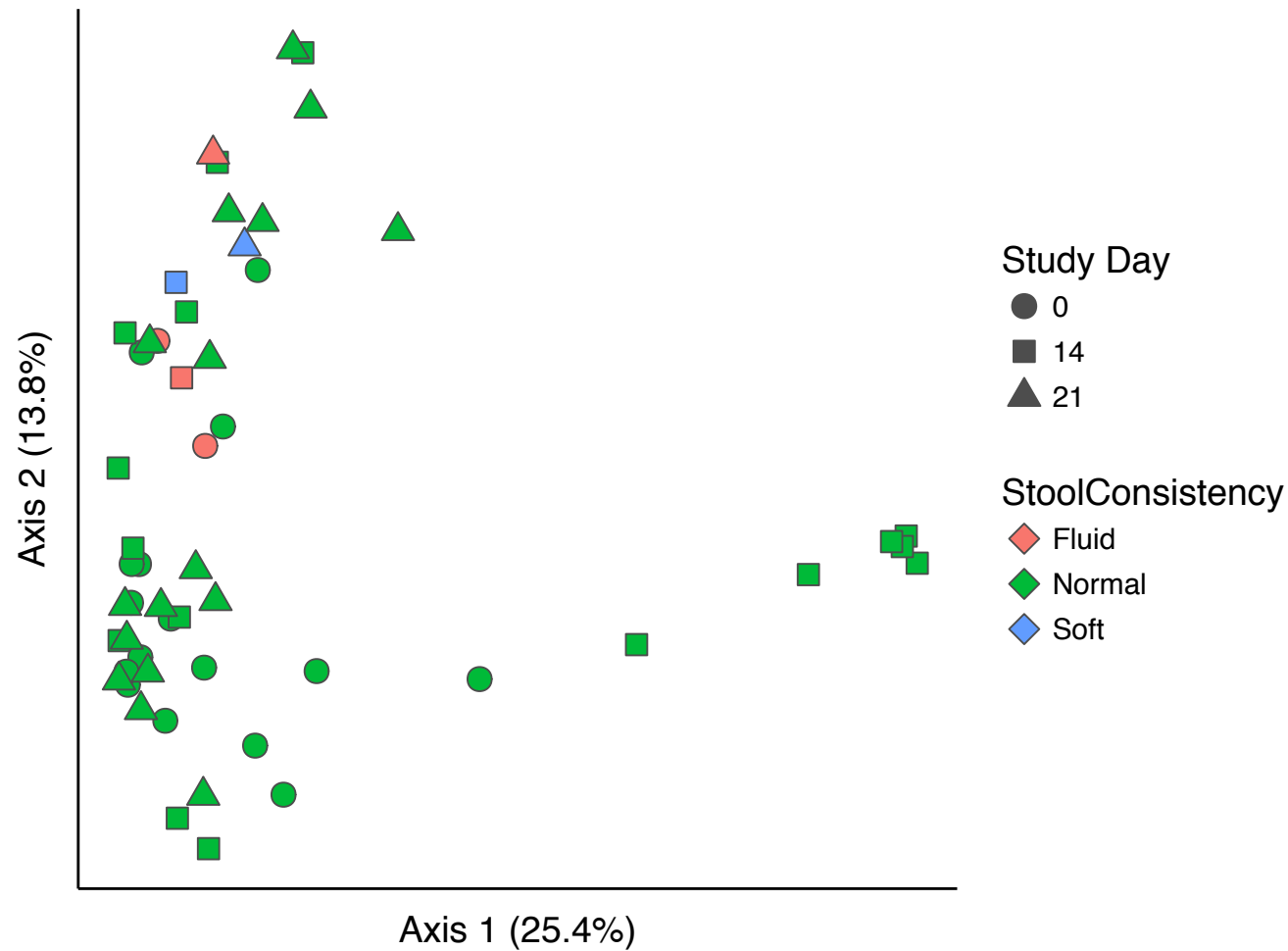

Supplement: Figure S3 — Principal coordinate decomposition (PCoA) plot showing breakdown of stool samples by consistency (color) and study day (shape). [file peerj-06-4612-s003.pdf]

Proportion

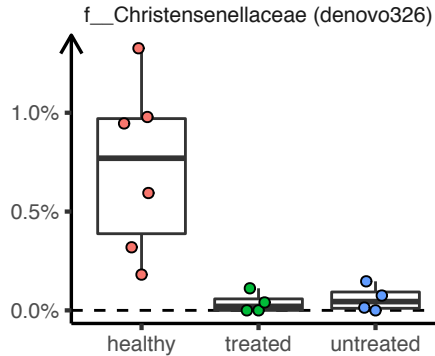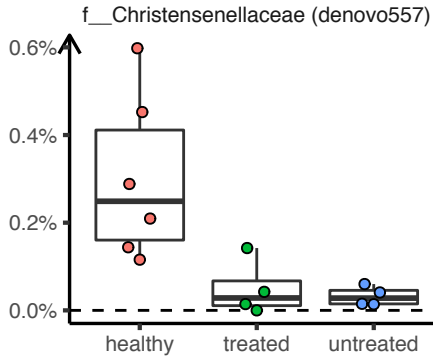

StudyGroup

- healthy
- treated
- untreated

Supplement: Figure S4 — The taxonomic assignment for each OTU is shown above the plot. [file peerj-06-4612-s004.pdf]

## Fungal taxa in feces; proportions

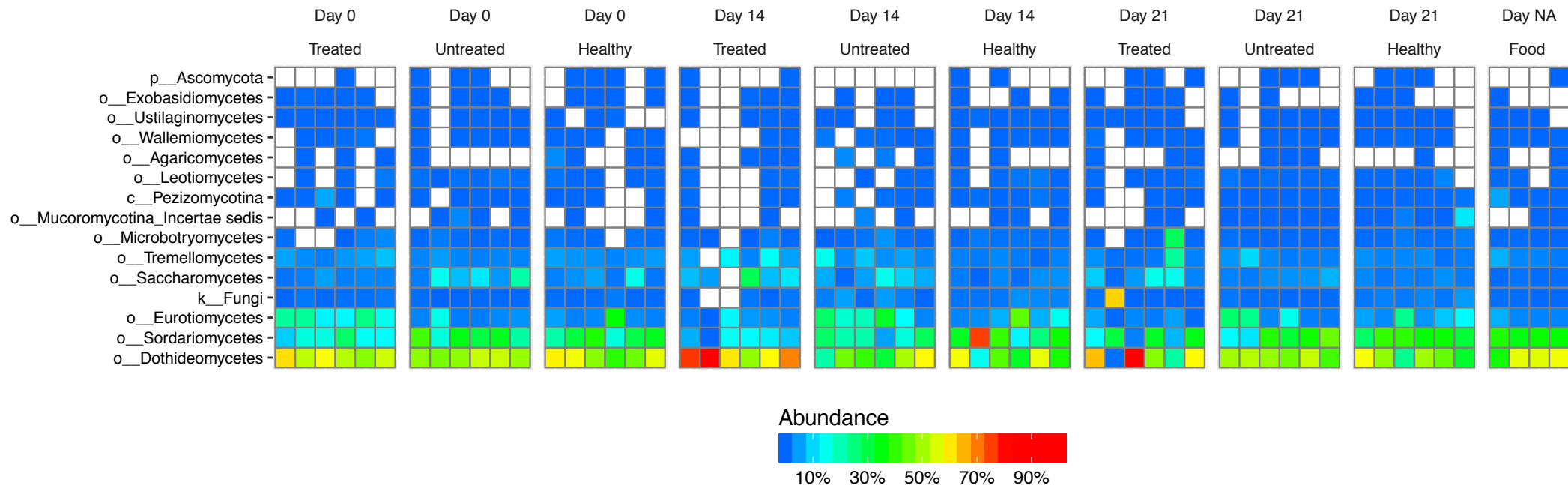

# B

## Fungal taxa in tissue; raw reads and blanks

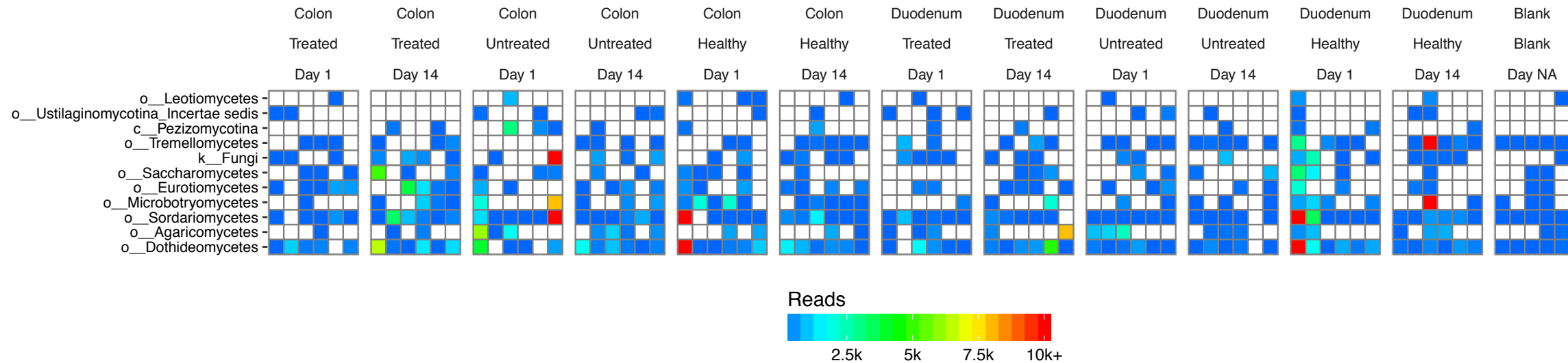

Supplement: Figure S5 — Alternative view of the fungal lineages shown in feces and tissue. In (A) the proportional abundance of each fungal taxa in feces is shown grouped first by study day and then by disease state. In (B) the major fungal taxa are shown in tissue samples alongside their proportion in the negative controls (blanks). [file peerj-06-4612-s005.pdf]

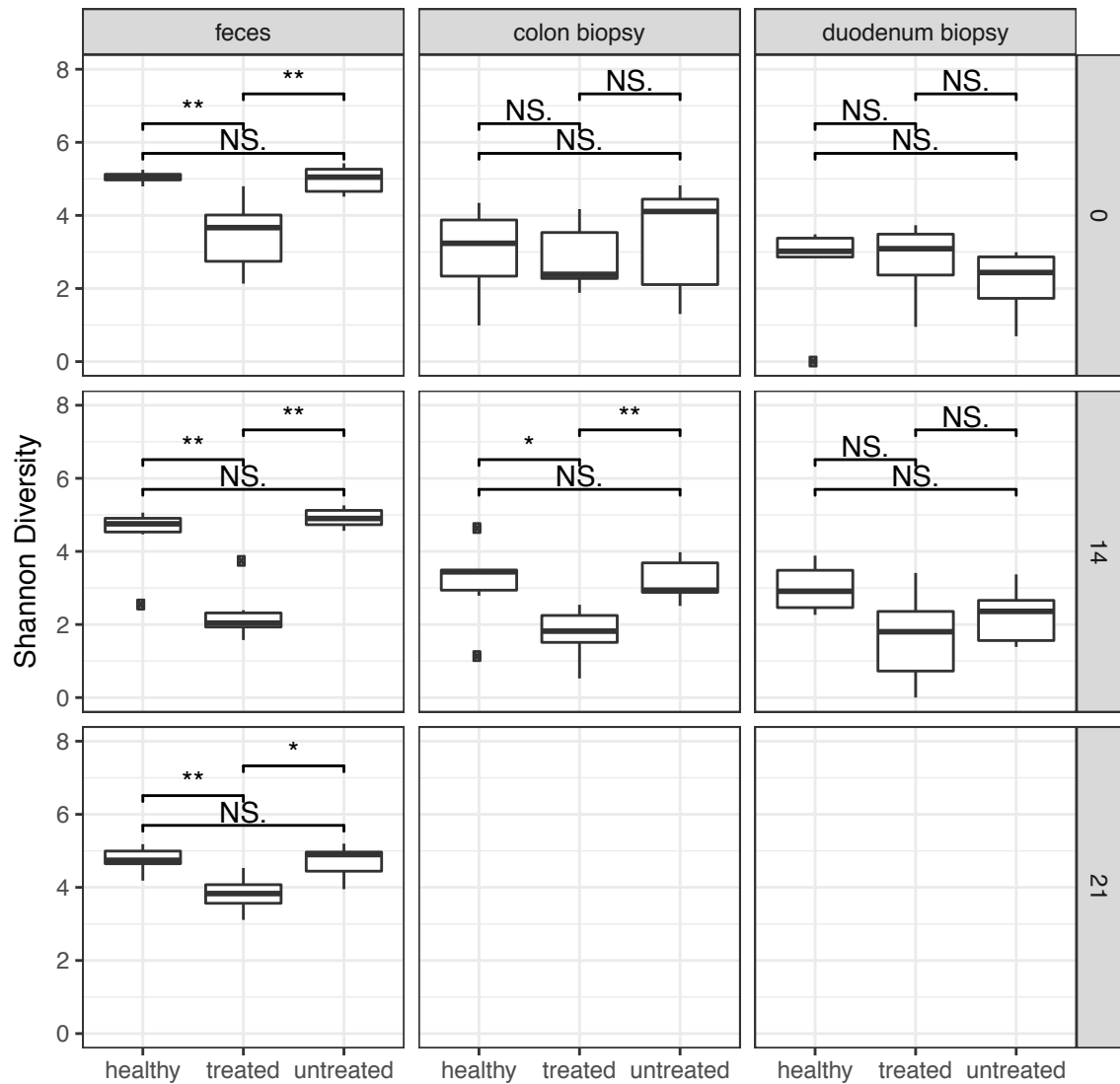

Supplement: Figure S6 — Each panel shows the bacterial diversity, measured by the Shannon index, of the three disease states (x-axis). The panels are separated by sample type (columns) and study day (rows). Each pairwise comparison was performed using the Wilcoxon rank-sum test and adjusted for multiple testing using FDR. *, p < 0.05; **, p < 0.01; NS, not significant. [file peerj-06-4612-s006.pdf]

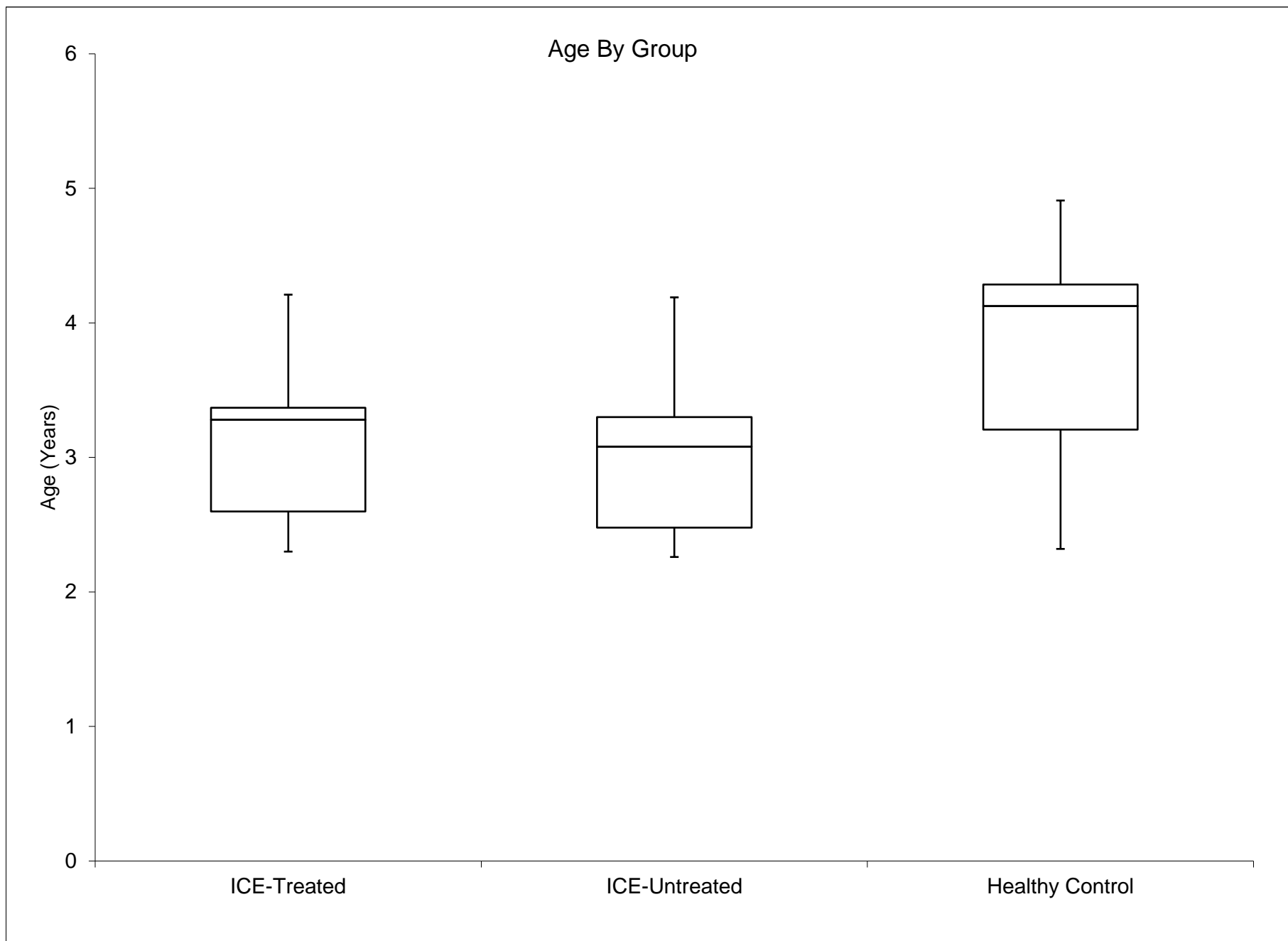

Supplement: Figure S7 — Box plots representing age (in years) distribution between groups. [file peerj-06-4612-s007.pdf]

Weights By Group

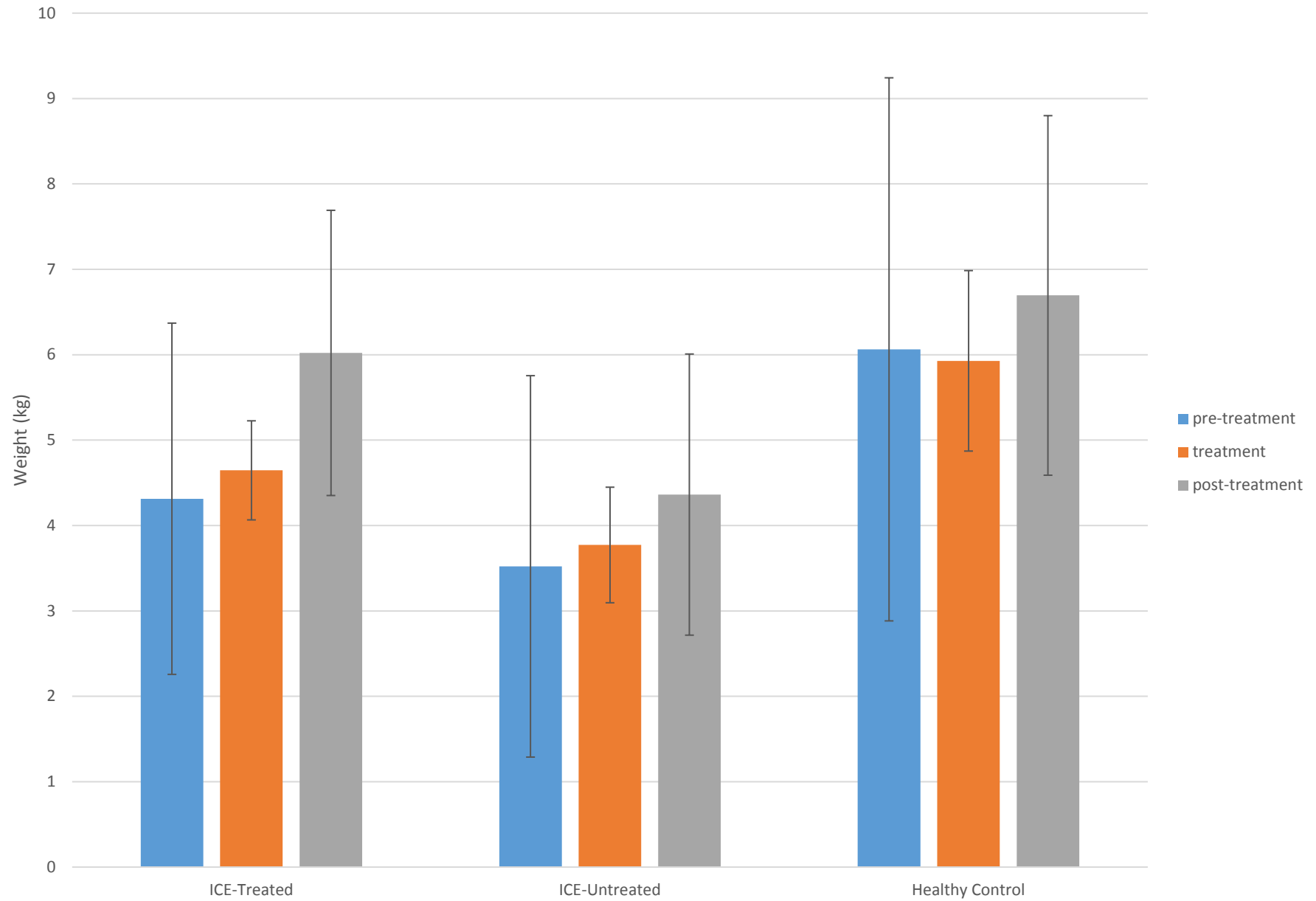

Supplement: Figure S8 — “Pre-treatment” values represent the mean of all weights during the 90-day pre-treatment observation period by group. “Treatment” values represent the mean of all weights during the 14-day treatment period by group. “Post-treatment” values represent the mean of all weights available during the 90-day post-treatment observation period by group. [file peerj-06-4612-s008.pdf]
